# GC-EI-MS datasets of trimethylsilyl (TMS) and *tert*-butyl dimethyl silyl (TBDMS) derivatives for development of machine learning-based compound identification approaches

**DOI:** 10.1016/j.dib.2023.109138

**Published:** 2023-04-11

**Authors:** Milka Ljoncheva, Sintija Stevanoska, Tina Kosjek, Sašo Džeroski

**Affiliations:** a Jozef Stefan Institute, Department of Environmental Sciences, Jamova 39, Ljubljana 1000, Slovenia; b Jozef Stefan Institute, Department of Knowledge Technologies, Jamova 39, Ljubljana 1000, Slovenia; c Jozef Stefan International Postgraduate School, Jamova 39, Ljubljana 1000, Slovenia

**Keywords:** Silylation, Derivative, Identification, Machine learning, GC-MS, Mass spectrometry

## Abstract

In the field of environment and health studies, recent trends have focused on the identification of contaminants of emerging concern (CEC). This is a complex, challenging task, as resources, such as compound databases (DBs) and mass spectral libraries (MSLs) concerning these compounds are very poor. This is particularly true for semi polar organic contaminants that have to be derivatized prior to gas chromatography-mass spectrometry (GC-MS) analysis with electron impact ionization (EI), for which it is barely possible to find any records. In particular, there is a severe lack of datasets of GC-EI-MS spectra generated and made publicly available for the purpose of development, validation and performance evaluation of cheminformatics-assisted compound structure identification (CSI) approaches, including novel cutting-edge machine learning (ML)-based approaches [1].

We set out to fill this gap and support the machine learning-assisted compound identification, thus aiding cheminformatics-assisted identification of silylated derivatives in GC-MS laboratories working in the field of environment and health. To this end, we have generated 12 datasets of GC-EI-MS spectra, six of which contain GC-EI-MS spectra of trimethylsilyl (TMS) and six GC-EI-MS spectra of *tert*-butyldimethylsilyl (TBDMS) derivatives. Four of these datasets, named testing datasets, contain mass spectra acquired by the authors. They are available in full, together with corresponding metadata. Eight datasets, named training datasets, were derived from mass spectra in the NIST 17 Mass Spectral Library. For these, we have only made the metadata publicly available, due to licensing reasons.

For each type of derivative, two testing datasets are generated by acquiring and processing GC-EI-MS spectra, such that they include raw and processed GC-EI-MS spectra of TMS and TBDMS derivatives of CECs, along with their corresponding metadata. The metadata contains IUPAC name, exact mass, molecular formula, InChI, InChIKey, SMILES and PubChemID, of each CEC and CEC-TMS or CEC-TBDMS derivative, where available. Eight GC-EI-MS training datasets are generated by using the National Institute of Standards and Technology (NIST)/U.S. Environmental Protection Agency (EPA)/National Institute of Health (NIH) 17 Mass Spectral Library. For each derivative type (TMS and TBDMS), four datasets are given, each corresponding to an original dataset obtained from NIST/EPA/NIH 17 and three variants thereof, obtained after each of the filtering steps of the procedure described below. Only the metadata about the training datasets are available, describing the corresponding NIST/EPA/NIH 17 entires: These include the compound name, CAS Registry number, InChIKey, exact mass, M_w_, NIST number and ID number.

The datasets we present here were used to train and test predictive models for identification of silylated derivatives built with ML approaches [4]. The models were built by using data curated from the NIST Mass Spectral Library 17 [2] and the machine learning approach of CSI:Output Kernel Regression (CSI:OKR) [2]. Data from the NIST Mass Spectral Library 17 are commercially available from the National Institute of Standards and Technology (NIST)/U.S. Environmental Protection Agency (EPA)/National Institute of Health (NIH) and thus cannot be made publicly available. This highlights the need for publicly available GC-EI-MS spectra, which we address by releasing in full the four testing datasets.

**Specifications Table**

**Table:** 

Subject	Analytical chemistry, Omics: General
Specific subject area	Generation of mass spectral datasets for testing and training of ML-based CSI approaches using mass spectra
Type of data	Raw and Table
How the data were acquired	The mass spectra in the testing datasets were acquired using Agilent 7890B/5977A series GC-MSD (Agilent Technologies, USA), in electron impact ionization (EI) mode. Chromatographic separation was achieved on Agilent DB-5MS UI fused-silica capillary column (30m x 0.25mm x 0.25 μm; Agilent
	Technologies, USA). Data was processed using Mass Hunter Quantitative Analysis v.B.07 (Agilent Technologies, USA). The training datasets were generated from the NIST/EPA/NIH Mass Spectral Library 17 [3] using the accompanying NIST Mass Spectral Search Program (version 2.3) and LIB2NIST converter (NIST, 2011).
Data format	The testing GC-EI-MS spectral datasets are given in .txt, .msp and .mgf formats and the accompanying metadata is given in .xlsx format. The metadata regarding the training GC-EI-MS spectral datasets is given in .xlsx format.
Description of data collection	The GC-EI-MS spectral datasets, that we used for testing ML-based CSI models, were generated in the full scan range of *m/z* 50-800 amu for the TMS derivatives and *m/z* 50-1000 amu for the TBDMS derivatives. Raw instrument data was reduced to two-dimensional peak lists (*m/z*, abundance) using Mass Hunter Qualitative Analysis v.B.07 (Agilent Technologies, USA), in which background subtraction was also performed. The GC-EI-MS spectral datasets intended for training of ML-based CSI models were generated from the NIST/EPA/NIH Mass Spectral Library 17: Constrained search was performed using the NIST Mass Spectral Search Program (version 2.3) and further processing according to the step-wise procedure described below.
Data source location	• *Institution: Jožef Stefan Institute, Department of Environmental Sciences* • *City/Town/Region: Ljubljana* • *Country: Slovenia*
Data accessibility	Repository name: Mendeley Data Data identification number: DOI:10.17632/j3z5bmvmnd.6 Direct URL to data: https://data.mendeley.com/datasets/j3z5bmvmnd/6
Related research article	Ljoncheva M., Stepišnik T., Kosjek T., Džeroski S., Machine learning for identification of silylated derivatives from mass spectra (2022) *Journal of Cheminformatics* 14(1):62. doi: 10.1186/s13321-022-00636-1.

## Value of the Data

• The generated GC-EI-MS datasets provide a comprehensive collection of GC-EI-MS spectra of TMS and TBDMS derivatives of structurally and chemically diverse environmental contaminants, given along with their metadata in universal ready-to-use formats (.txt, .msp, .mgf) for further cheminformatics-based processing.
• The generated GC-EI-MS datasets are of value for the environmental and exposomics researchers, as well as for the CSI and ML communities, interested in the development of new CSI tools.
• Few datasets of mass spectra are publicly available: This is especially true for GC-EI-MS spectra, which makes the generated data even more valuable.
• Both the testing and the training data can be further used on their own or as part of larger datasets, for training, testing and validation in the development of novel CSI approaches, for challenging existing approaches, and for performance comparison of novel and existing CSI, especially ML-based approaches.
• The data can be used as a stand-alone database (or joined with other in-house databases of GC-EI-MS spectra), serving as valuable reference during suspect screening and non-targeted environmental analysis.

## 1 Introduction

NIST/EPA/NIH 17 Mass Spectral Library [3] was used to generate training GCI-EI-MS spectral datasets of TMS and TBDMS derivatives, which are then used for building ML-based CSI approaches. As NIST/EPA/NIH 17 Mass Spectral Library [3] is commercially available and licensed under the United States Department of Commerce Copyright, the training GC-EI-MS spectral datasets themselves cannot be made publicly available. Instead, we provided metadata files of each dataset, containing the name, InChIKey, CAS Registry number, exact mass, molecular weight (M_w_), NIST number and ID number for each GC-EI-MS spectrum. For each derivative type (TMS and TBDMS), four GC-EI-MS datasets were generated – the first ones (TMS_0.1 and TBDMS_0.1) containing the GC-EI-MS spectra initially extracted from NIST/EPA/NIH 17 Mass Spectral Library (“Metadata_training_TMS_0.1”, “Metadata_ training_TBDMS_0.1”), followed by TMS_1.3 and TBDMS_1.3 resulting from the first filtering step of the approach described below (“Metadata_ training_TMS_1.3”, “Metadata_ training_TBDMS_1.3”), TMS_2.3 and TBDMS_2.3, resulting from the second filtering step (“Metadata_ training_TMS_2.3”, “Metadata_ training_TBDMS_2.3”), and TMS_3.3 and TBDMS_3.3, resulting from the final, third filtering step (“Metadata_training_TMS_3.3”, “Metadata_ training_TBDMS_3.3”). Using the given metadata and the described procedure (described in more details by Ljoncheva et al. [2]), the training GC-EI-MS spectral datasets can be reconstructed.

Standard solutions of selected environmental contaminants (104 compounds), listed in Table 1, were used for generating the TMS and TBDMS test dataset. The presented data consists of .txt, .msp and .mgf data files for each of the four MS datasets (Test dataset_TMS_RAW, Test dataset_TMS_BS, Test dataset_TBDMS_RAW and Test dataset_TBDMS_BS), in which each of the GCRC-EI-MS spectra is recorded with the compound name, InChIKey, M_w_, molecular formula (MF), CAS Registry number and list of peaks represented as *m/z* and intensities. Metadata of the TMS/TBDMS derivatives and the corresponding parent CEC are given in .xlsx files containing the IUPAC name, exact mass, MF, InChI, InChIKey, SMILES and PubChem ID, when available (“Metadata_test_TMS derivatives.xlsx” and “Metadata_test_TBDMS derivatives.xlsx”). The four datasets were used to test predictive models for identification of silylated derivatives, built with ML approaches.

Note that for some of the spectra in the test datasets, spectra of the corresponding compounds also appear in the training datasets. Evaluation of the models generated by ML has been conducted separately on spectra of compounds whose spectra appear/do not appear in the training datasets, respectively, and results are reported separately by Ljoncheva et al. [2]. In the metadata files for the testing datasets (“Metadata_test_TMS derivatives.xlsx” and “Metadata_test_TBDMS derivatives.xlsx”), an extra column (the last one) indicates whether a spectrum of the compound at hand also appears in the corresponding training dataset and the respective metadata file (“Metadata_training_TMS_3.3”, “Metadata_ training_TBDMS_3.3”).

The predictive models for CSI of silylated derivatives were built by ML approaches from training datasets of GC-EI-MS spectra of TMS and TBDMS derivatives, which are not publicly available, as they were curated from the commercially available NIST/EPA/NIH Mass Spectral Library 17 [3], licensed under the United States Department of Commerce Copyright. NIST's end-user's license for the NIST 17 MSL restricts its use to a single computer that is not accessible by more than one person. While the training datasets themselves cannot be made publicly available, we make available the corresponding metadata: With licensed address to NIST MSL 17, they can be used to reconstruct the training datasets, by following the workflow summarized below and described by Ljoncheva et al. [2].

The ML approach used to build models for the identification of silylated derivatives from these data [2] was the approach titled CSI:OKR [3].

## 2 Experimental Design, Materials and Methods

### 2.1 Experimental design and generation of training datasets

Initial versions of the TMS and TBDMS datasets (TMS_0.1 and TBDMS_0.1) were generated by extracting all GC-EI-MS spectra of TMS, resp. TBDMS, derivatives of small molecules from the NIST/EPA/NIH 17 Mass Spectral Library [3]. The first constrained search for GC-EI-MS TMS spectra, using the constraints *name fragment: trimethylsilyl* and *elements allowed: Si*, resulted in a collection of 9958 entries, while for GC-EI-MS TBDMS spectra, the constraints *name fragment: tertbutyldimethylsilyl* and *elements allowed: Si, r*esulted in an initial dataset of 2238 entries. Entries were extracted in .msp file format and subsequently converted to .txt format, using the LIB2NIST conversion tool (NIST 2011). Each GC-EI-MS entry included the compound name, InChIKey, MF, M_w_, exact mass, CAS number, NIST ID and MS peak list. The GC-EI-MS spectra of TMS/TBDMS derivatives with erroneous metadata (name, molecular formula, InChIKey) that do not correspond to the analyzed compound were excluded from the dataset.

The TMS/TBDMS GI-EI-MS spectral datasets were further filtered using a three-step spectral filtering process, including:

1) Exclusion of chemical irregularities. The GC-EI-MS spectra of TMS, resp. TBDMS derivatives of compounds not susceptible to derivatization, defined by the absence of functional group(s) amenable to silylation, were filtered out. The functional groups amenable to silylation are those containing an active hydrogen, i.e., carboxyl, hydroxyl, amine and thiol.
2) Exclusion of high-molecular mass TMS, resp. TBDMS derivatives. The GC-EI-MS spectra of TMS, resp. TBDMS derivatives of CEC with molecular mass ≥ *m/z* 1000 were eliminated, since, as such, they are above the working linear range of the GC-MS instruments.
3) Exclusion of insufficient-quality GC-EI-MS spectra. The following GC-EI-MS spectra were excluded:
  - GC-EI-MS spectra not acquired at the upper *m/z* of at least M_w_ of the derivative + 10 amu;
  - GC-EI-MS spectra that do not contain both the molecular ion [M]^+^ peak and at least one of the isotope peaks, such as the ^13^C isotope peak;
  - GC-EI-MS spectra that contain neither peaks of fragment ions specific for TMS groups (*m/z* 73, 147, 221 and 295, corresponding to one, two, three and four TMS groups, respectively) nor for TBDMS groups (*m/z* 115, 230 and 345, corresponding to one, two and three TBDMS groups, respectively) and
  - GC-EI-MS spectra not containing at least five fragment ion peaks.

As a result, the final version of the TMS dataset consists of 4648 TMS GC-EI-MS spectra, while the final version of the TBDMS dataset consists of 1883 GC-EI-MS spectra. For each of the GC-EI-MS spectra in the final TMS and TBMDS datasets, the *m/z* range was between 50 *m/z* to M_w_ of the derivative ± 10 amu. For data parsing, all ion fragments with intensity 0 were removed from the refined TMS and TBDMS datasets.

### 2.2 Chemical analysis for generation of test datasets

#### 2.2.1 Chemicals and materials

From the in-house pool of reference standards, 104 CEC were selected as environmentally relevant, according to the criteria of the Regulation (EC) No.1907/2006 of the European Parliament and the Council of 18 December 2006 concerning the Registration, Evaluation, Authorisation and Restriction of Chemicals (REACH), Annex III [4]. These are listed in Table 1.

**Table 1:** CEC for generation of the TMS and TBDMS datasets.

CEC included in TMS datasets only	

nitroxoline 17α-hydroxyprogesterone 6β-hydroxypregnenolone 5-androstene-3β, 17β-diol 5α-dehydrotestosterone (boldenone) 11α-hydroxytestosterone 11α-hydroxyandrostenedione methamphetamine nylidrin (-)-quinic acid	shikimic acid meso-erythritol amphetamine 6-nitroguaiacol estriol codeine L-tyrosine L-ascorbic acid cannabidiolic acid bisphenol FL butylated hydroxytoluene

CEC included in TBDMS datasets only	

4,6-dinitroguaiacol	mycophenolic acid

CEC included in both TMS and TBDMS datasets	

bisphenol A benzoic acid mecoprop 4,4’-biphenol 4,4′-dihydroxydiphenyl ether 4,4′-isopropylidenebis(2,6-dimethylphenol) 2,4′-dihydroxydiphenylmethane (24BPF) bisphenol AF bisphenol AP bisphenol C bisphenol E bisphenol F bisphenol M bisphenol BP bisphenol P bisphenol S bisphenol Z 2,2′-methylenediphenol (22BPF) dihydrotestosterone (stanolone) (±)-11-hydroxy-Δ^9^- tetrahydrocannabinol (±)-11-nor-9-carboxy-Δ^9^-tetrahydrocannabinol sulfanilamide adipic acid 4-tert-octylphenol 9-hydroxyfluorene L-leucine L-serine (-)-Δ9 tetrahydrocannabinol (-)-Δ9 tetrahydrocannabinolic acid trans-3’-hydroxycotinine benzoylecgonine bisphenol CL bisphenol PH 8-hydroxyquinoline 2-anilinophenylacetic acid 4-nitroguaiacol 5-nitroguaiacol catechol 3-methylcatechol 3-methyl-5-nitrocatechol	2-benzyl-4-chlorophenol citric acid monohydrate 4-cumylphenol 2,4-dihydroxybenzophenone estrone 17β-estradiol m-coumaric acid p-coumaric acid o-coumaric acid triclosan (+)-cannabidiol cannabinol cannabichromene morphine 6-monoacetylmorpine carbamazepine isopropylparaben bisphenol B 17α-ethynyl estradiol 4-hydroxybenzophenone 2,2′-dihydroxy-4-methoxybenzophenone (BP-8) clofibric acid ibuprofen naproxen ketoprofen diclofenac methylparaben ethylparaben propylparaben butylparaben isobutylparaben benzylparaben 4-nonylphenol phenylacetic acid resorcinol salicylic acid urea 4-nitrocatechol syringol 4-nitrosyringol etofylline

The selected compounds had to satisfy at least three of the following five criteria: **1) Positioning**: the compound is present in the US EPA Comptox Chemistry Dashboard (CCD) [5], the most comprehensive repository of EE constituents; **2) Persistence**: compound's half-life in fresh or estuarine water > 40 days; **3) Bioaccumulation**: BAF and/or BCF > 2000, or in absence of such data, logK_ow_ ≥ 5.0; **4) Mobility**: compound's water solubility ≥ 0.15 mg/L and log K_oc_ ≤ 4.0, i.e. between -10.0 and 4.0; and **5) EcoToxicity**: long-term no-observed-effect concentration (NOEC) for marine or freshwater organisms < 0.01 mg/L. Further details of the selection procedure are given by Ljoncheva et al. [2].

Individual stock solutions of each CEC at a concentration of approximately 150 μg/mL were prepared in acetonitrile (ACN), ethyl acetate (EtAc) or methanol (MeOH), depending on CEC solubility. Of them, individual working solutions (IWS) at concentration 1 µg/mL were prepared and used within 7 days. The CEC included in each GC-EI-MS dataset are listed in Table 1.

#### 2.2.2 Derivatization and analysis

For each CEC, to 500 µL of IWS, 470 µL EtAc and 30 µL derivatization agent was added; for generation of TMS derivatives, 30 µL N, O-bis trifluoroacetamide with 1% trimethylchlorosilane (BSTFA + 1% TMCS), for TBDMS derivatives N-*tert*-butyldimethylsilyl-N-methyltrifluoroacetamide (MTBSTFA) with 1% TMCS (MTBSTFA + 1% TMCS). For CECs whose IWS are in ACN or MeOH, the 500 µL IWS was dried under N_2_ flow, reconstituted in 970 µL EtAc, to which 30 µL of the appropriate derivatization agent were added at predefined reaction temperature and duration. 106 TMS derivatives of 102 CEC (4 CEC resulted in two TMS derivatives, namely salicylic acid, dihydrotestosterone (stanolone), sulfanilamide and 5α-androsten-3β, 17β-diol) and 85 TBDMS derivatives of 83 CEC, two with two TBDMS derivatives each (sulfanilamide and L-serine) were generated, with molecular weights of derivatives ranging up to 650 amu.

GC-EI-MS spectra were acquired on Agilent 7890B/5977A series GC-MSD (Agilent Technologies, USA). Separation was achieved on Agilent DB-5MS UI fused-silica capillary column (30 m x 0.25 mm x 0.25 μm; Agilent Technologies, USA). He of 99.99999% purity at the flow rate of 1.2 mL/min was used as a carrier gas. The manifold, ion source and transfer line temperatures were set at 230°C, 150°C and 250°C, respectively. Injections (1 µL) were performed in the splitless mode. Depending upon compound properties, one of the following column oven temperature programs was used for generation of GC-EI-MS spectra of TMS derivatives: (1) initial temperature 70 °C (held 1 min), ramped at 15 °C/min to 280 °C (held 1 min); total runtime: 16 min; (2) initial temperature 70 °C (held 1 min), ramped at 20 °C/min to 240 °C (held 1 min), at 12 °C/min to 310 °C (held 2 min); total runtime: 18.3 min; (3) initial temperature 70 °C (held 1 min), ramped at 20 °C/min to 240 °C (held 1 min), at 12 °C/min to 310 °C (held 4 min); total runtime: 20.3 min. For generation of GC-EI-MS spectra of TBDMS derivatives, the following column oven temperature program was used: initial temperature 70 °C (held 1 min), ramped at 10 °C/min to 240 °C (held 1 min), at 10°C/min to 310 °C (held 5 min); total runtime: 31 min.

The MSD was operated in EI ionization mode (70 eV) by scanning over the mass range of *m/z* 50-800 amu for TMS derivatives and *m/z* 50-1000 amu for TBDMS derivatives. In-between the acquisitions of the derivatized standards, EtAc was run as the solvent check to assess potential background interferences and was used for background subtraction as a part of the post-acquisition processing of the GC-EI-MS spectra.

The retention times (R_t_) of the TMS and TBDMS derivatives are given in Table 2 and Table 3, respectively.

**Table 2:** R_t_s of CEC-TMS derivatives.

TMS derivative	R_t_ (min)	TMS derivative	R_t_ (min)
*GC-MS acquisition method (1)*			

benzoic acid TMS	6.669	propylparaben TMS	10.300
methylparaben TMS	8.930	isobutylparaben TMS	10.730
salicylic acid-bis TMS	9.020	butylparaben TMS	11.080
ethylparaben TMS	9.520	shikimic acid TMS	11.260
isopropylparaben TMS	9.760	quinic acid TMS	11.620
ibuprofen TMS	9.960	triclosan TMS	13.560
mecoprop TMS	10.040	benzylparaben TMS	13.840
		cannabidiol TMS	14.200

*GC-MS acquisition method (2)*			

resorcinol TMS	6.750	carbamazepine TMS	12.360
clofibric acid TMS	8.070	diclofenac TMS	12.530
9-hydroxyfluorene TMS	9.440	cannabichromene TMS	12.805
4-cumylphenol TMS	9.883	Δ^9^-tetrahydrocannabinol TMS	13.041
naproxen TMS	11.070	cannabinol TMS	13.630
4-nonylphenol TMS	10.010	Δ^9^-tetrahydrocannabinolic acid TMS	14.882
2,4-dihydroxybenzophenone TMS	11.130	estrone 2TMS	14.882
4,4′-biphenol 2TMS	11.490	estradiol TMS	15.083
ketoprofen TMS	11.510	ethinylestradiol TMS	15.142
sulfanilamide 2TMS	11.910	estriol 3TMS	16.145
2,2′-dihydroxy-4-methoxybenzophenone TMS	12.108		

*GC-MS acquisition method (3)*			

phenylacetic acid TMS	6.203	bisphenol E 2TMS	11.692
		bisphenol A 2TMS	11.874
catechol 2TMS	6.329	L-serine TMS	11.902
L-serine 3TMS	6.571	bisphenol C 2TMS	11.932
syringol TMS	6.908	bisphenol B 2TMS	12.462
3-methtylcatechol TMS	6.928	benzoylecgonine TMS	12.582
urea 2TMS	7.124	methamphetamine TMS	12.590
erythritol 4TMS	7.542	4,4′-isopropylidenebis(2,6-dimethylphenol) TMS	13.432
adipic acid TMS	7.632	bisphenol CL 2TMS	13.571
8-hydroxyquinoline TMS	7.803	nylidrin TMS	13.579
6-nitroguaiacol TMS	8.202	codeine TMS	13.811
4-octylphenol TMS	8.450	morphine 2TMS	14.112
4-nitroguaiacol TMS	8.623	bisphenol Z 2TMS	14.462
5-nitroguaiacol TMS	8.728	6-monoacetylmorphine TMS	14.562

4-nitrocatechol TMS	9.044	5-androstene-3β,17β-diol TMS	14.599
citric acid TMS	9.330	quinic acid TMS	14.623
p-coumaric acid TMS	9.359	5-androstene-3β,17β-diol 2TMS	14.641

4-nitrosyringol TMS	9.475	11-hydroxytetrahydrocannabinol 2TMS	14.652
3-methyl-5-nitrocatechol TMS	9.538	stanolone TMS	14.757
L-leucine TMS	9.629	stanolone 2TMS	14.873
m-coumaric acid TMS	9.728	bisphenol S 2TMS	14.971
butylated hydroxytoluene TMS	9.722	bisphenol AP 2TMS	15.183
trans-3’-hydroxycotinine TMS	9.842	boldenone TMS	15.420
clorophene TMS	10.002	11-nor9-tetrahydrocannabinol 2TMS	15.553
o-coumaric acid TMS	10.085	L-tyrosine TMS	15.704
nitroxoline TMS	10.149	L-ascorbic acid TMS	15.846
2,2-bisphenol F 2TMS	10.231	11α-hydroxyandrostenedione TMS	15.988
bisphenol AF TMS	10.492	11α-hydroxytestosterone TMS	16.188
amphetamine TMS	10.551	bisphenol M 2TMS	16.193
2,4-bisphenol F 2TMS	10.863	6β-hydroxypregnenolone TMS	16.693
2-anilinophenylacetic acid TMS	10.885	17α-hydroxyprogesterone TMS	17.135
2,4-dihydroxybenzophenone TMS	11.132	bisphenol P 2TMS	17.242
shikimic acid TMS	11.271	bisphenol BP 2TMS	17.695
etofylline TMS	11.253	bisphenol PH 2TMS	17.832
4,4′-dihydroxydiphenyl ether 2TMS	11.423	cannabidiolic acid TMS	18.269
bisphenol F 2TMS	11.512	bisphenol FL 2TMS	18.902

**Table 3:** R_t_s of CEC-TBDMS derivatives.

TBDMS derivative	R_t_ (min)	TBDMS derivative	R_t_ (min)
benzoic acid TBDMS	8.139	benzoylecgonine TBDMS	17.724
benzeneacetic acid TBDMS	8.433	cannabichromene TBDMS	17.851
methylparaben TBDMS	10.327	citric acid TBDMS	17.956
8-hydroquinone TBDMS	10.738	tetrahydrocannabinol TBDMS	18.040
clofibric acid TBDMS	11.011	3,4-dehydroTBDMS	18.093
ethylparaben TBDMS	11.032	cannabidiol TBDMS	18.566
resorcinol TBMDS	11.074	DHDPE TBDMS	18.587
isopropylparaben TBDMS	11.316	bisphenol F TBDMS	18.619
ibuprofen TBDMS	11.401	bisphenol E TBDMS	18.756
mecoprop TBDMS	11.506	4,4’-bisphenol TBDMS	18.787
4-tertoctylphenol TBDMS	11.727	bisphenol 8 TBDMS	18.798
propylparaben TBDMS	12.032	cannabinol TBDMS	18.819
salicylic acid TBDMS	12.379	bisphenol A TBDMS	18.977
adipic acid TBDMS	12.442	morphine TBDMS	19.134
isobutylparaben TBDMS	12.590	bisphenol B TBDMS	19.566
butytlparaben TBDMS	13.063	bipshenol C TBDMS	19.639
4-nonylphenol TBDMS	14.462	6-monoacetylmorphine TBDMS	20.008

9-hydroxyfluorene TBDMS	13.463	estrone TBMDS	20.534
erythritol TBDMS	13.610	bisphenol CL TBDMS	20.839
trans-3’-hydroxycotinine TBDMS	14.042	BP26DM TBDMS	20.860
4-cumylphenol TBDMS	14.273	11-hydroxytetrahydrocannabinol TBDMS	21.144
chlorophene TBDMS	14.389	ethinyl estradiol TBDMS	21.144
4-hydroxybenzophenone TBDMS	15.725	tetrahydrocannabinolic acid TBDMS	21.249
naproxen TBDMS	15.757	bisphenol Z TBDMS	21.733
tricolsan TBDMS	16.199	11-nor-9-tetrahydrocannabinol TBDMS	22.154
sulfanilamide TBDMS	16.399	bisphenol S TBDMS	22.607
2,2-bisphenol F TBDMS	16.578	bisphenol AP TBDMS	22.649
ketoprofen TBDMS	16.883	estradiol TBDMS	23.059
benzylparaben TBDMS	16.925	bisphenol M TBDMS	24.185
bisphenol AF TBDMS	17.114	bisphenol P TBDMS	26.479
carbamazepine TBDMS	17.356	bisphenol PH TBDMS	27.079
2.4-bisphenol F TBDMS	17.630	bisphenol BP TBDMS	27.457
diclofenac TBDMS	17.693		

### 2.3 Data processing

GC-EI-MS data acquisition resulted in the generation of multiple (≥15) GC-EI-MS spectra for most of the TMS and TBDMS derivatives. Exceptions are the L-ascorbic acid TMS, L-leucine TMS and L-serine TMS, with three GC-EI-MS spectra each, and the TBDMS derivatives of L-serine, 4-nitroguaiacol, 5-nitroguaiacol, catechol, 3-methylcatechol, 3-methyl-5-nitrocatechol, syringol, 4-nitrosyringol, 4-nitrocatechol, p-coumaric acid, m-coumaric acid, o-coumaric acid, mycophenolic acid, 4,6-dinitroguaiacol, etofylline and urea, with one GC-EI-MS spectrum in the test TBDMS datasets for each. All GC-EI-MS spectra were processed using Mass Hunter Qualitative Analysis v B.07 (Agilent Technologies, USA) that reduced raw instrument data to two-dimensional peak lists (*m/z*, abundance), exported in .txt format. This software was also used to perform background subtraction, in order to remove constantly present background signals, such as *m/z* 149 as a typical phtalate interference, *m/z* 282, *m/z* 256 and *m/z* 284 for oleic, palmitic and stearic acid, and *m/z* 207, *m/z* 281 and *m/z* 327 of common polysiloxanes resulting from GC column stationary phase degradation. Their presence was confirmed *a priori* in the multiple EtAc solvent runs acquired between the acquisitions of CEC silyl derivatives, and was used for background subtraction.

The .txt data were transformed into .mgf format by a Python script which formats the beginning of a new spectrum in .mgf required syntax (i.e., ``BEGIN IONS''), then lists the exact mass of the compound (e.g., ``MASS=194.076''), taken from the appropriate file with the metadata, followed by a row with the charge (``CHARGE=1+''). The introductory part of the data record finishes with a line starting with ``TITLE= InChIKey:'', followed by the InChIKey of the compound, then “Name:” and the name of the compound, where the InChIKey and the name of the compound are taken from the .txt file, followed by an empty line. The peaks are then copied verbatim from the .txt file, line after line, and, afterwards, the data record finishes with the row "END IONS", preceded by an empty line. Each spectrum entry from the .txt files is thus converted into .mgf format and the spectra are listed in the same order as in the .txt file.

The .mgf files can be read by the ProteoWizard MSConvert software (version 3.0.22153-da6d3d1) [6] and consequently converted into a number of other formats that are in use by the MS community (such as mzML or mzXML). Unfortunately, these formats do not include the .msp format. The .msp format of the data was generated from the above described .mgf format by using the Python library available at https://github.com/matchms/matchms.

## Ethics Statements

The authors declare that the manuscript meets all the rules and conditions described in the “Ethics in publishing” section standards (https://www.elsevier.com/journals/data-in-brief/2352-3409/guide-for-authors). The work did not include any investigations involving animal experiments, human participants and data collected from social media platforms.

The training GC-EI-MS spectral datasets were curated from the commercially available NIST/EPA NIH 17 Mass Spectral Library. Explicit permission to release the metadata about the training datasets was obtained by the authors from NIST. Due to NIST's individual license, restricting the use to a single computer that is not accessible by more than one person, the training datasets cannot be made available to the public by the authors. However, with licensed access to the NIST 17 MSL, the training data can be reconstructed from the available metadata files by following the detailed description of data preparation, given above.

## CRediT authorship contribution statement

**Milka Ljoncheva:** Investigation, Formal analysis, Data curation, Software, Writing – original draft, Writing – review & editing. **Sintija Stevanoska:** Data curation, Software. **Tina Kosjek:** Conceptualization, Resources, Validation, Supervision, Writing – review & editing. **Sašo Džeroski:** Conceptualization, Resources, Validation, Supervision, Writing – review & editing.

## Declaration of Competing Interest

The authors declare that they have no known competing financial interests or personal relationships that could have appeared to influence the work reported in this paper.

## Data Availability

GC-EI-MS datasets of trimethylsilyl (TMS) and tert-butyl dimethylsilyl (TBDMS) derivatives for development of machine learning-based compound identification approaches (Original data) (Mendeley Data).
